# Labral morphology does not compensate for reduced bony glenoid concavity in stable shoulders

**DOI:** 10.1016/j.jseint.2025.101422

**Published:** 2025-12-16

**Authors:** Alexander J. Vervaecke, Charles Thery, Victor Housset, Philipp Moroder, Jean-David Werthel

**Affiliations:** a Hôpital Ambroise-Paré, Boulogne Billancourt, France; b Monica Orthopaedic Research (MoRe) Foundation, Antwerp, Belgium; c Orthopaedic Center Antwerp (Orthoca), AZ Monica, Antwerp, Belgium; d Schulthess Klinik, Zurich, Switzerland

**Keywords:** Shoulder instability, Shoulder surgery, Shoulder dislocation, Glenoid concavity, Labrum, Glenoid bone loss

## Abstract

**Background:**

Glenoid concavity plays a critical role in shoulder stability via the concavity-compression mechanism. While the bony glenoid concavity, quantified by the bony shoulder stability ratio (BSSR), is a known determinant of stability, the labrum also contributes to the overall glenoid concavity. It remains unclear whether the labrum compensates for reduced bony concavity in stable shoulders. This study aimed to investigate the relationship between labral and bony glenoid concavity implementing the labral shoulder stability ratio (LSSR) and BSSR, respectively. We hypothesized that shoulders with reduced bony concavity (lower BSSR) would demonstrate increased labral concavity (higher LSSR), suggesting a compensatory mechanism.

**Methods:**

In this retrospective imaging study, 36 patients (mean age: 26.7 years) undergoing shoulder computed tomography arthrography between January 2020 and December 2024 for noninstability indications were included. BSSR and LSSR were calculated from standardized axial computed tomography images using three-dimensional multiplanar reconstructions. Concavity depth and radius were measured on the bony and chondrolabral contours, and the respective stability ratios were calculated. Inter-rater reliability was assessed using Bland-Altman plots and Pearson correlation. Pearson correlation analysis and subgroup comparisons were conducted to assess the relationship between BSSR and LSSR.

**Results:**

The mean BSSR was 28.3% ± 11.1% (range: 10.2%-52.5%), and the mean LSSR was 77.9% ± 10.8% (range: 49.1%-100%). There was no significant correlation between BSSR and LSSR (r = 0.01, *P* = 1.000). A low positive correlation was observed between glenoid bone depth and labral depth (r = 0.33, *P* = .049), and no significant relationship was found between the radius of the bony and labral best-fit circles (r = −0.11, *P* = .515). Subgroup analysis comparing patients with low BSSR (≤25th percentile) vs. high BSSR (≥75th percentile) showed no significant difference in LSSR values (78.8% vs. 75.9%, *P* = .554). Inter-rater agreement was good for both BSSR and LSSR measurements.

**Conclusion:**

This study demonstrates that labral morphology does not compensate for reduced bony glenoid concavity in clinically stable shoulders. Contrary to our hypothesis, lower BSSR was not associated with increased labral concavity, and no inverse relationship was observed between bone and labral curvature.

Shoulder stability relies on a complex interplay of static and dynamic stabilizers, including the bony glenoid concavity, soft tissue structures such as the labrum, and dynamic stabilizers like the rotator cuff.^7^^,^^22^ The concavity-compression mechanism describes the biomechanical synergy by which the rotator cuff compresses the convex humeral head into the concave glenoid fossa, centering it and stabilizing the joint, particularly in midrange motion where capsuloligamentous laxity is greatest.^8^^,^^13^ This mechanism depends critically on the three-dimensional morphology of the glenoid concavity, incorporating contributions from bone, cartilage, and labrum.^14^

The glenoid bony concavity and curvature significantly determines glenohumeral congruency, directly influencing shoulder stability. Moroder et al^15^^,^^16^ introduced the concept of the bony shoulder stability ratio (BSSR), a quantifiable measure reflecting the relationship between bony glenoid concavity depth and radius. Their work demonstrated a strong correlation between increased glenoid concavity and anterior stability, highlighting the inadequacy of conventional one-dimensional or two-dimensional defect size assessments for evaluating glenoid bone loss. Notably, they showed that the biomechanical impact of a bony defect depends not only on its size but also on intraindividual variations in glenoid concavity. Building on this, recent work by Oenning et al^19^ demonstrated that highly concave glenoids can tolerate up to 20% glenoid bone loss while maintaining the same level of stability as native flat glenoids with low concavity. These findings underscore the pivotal role of glenoid concavity in preserving shoulder stability, particularly in the context of bone loss.^20^

The glenoid labrum enhances the glenoid concavity and cocontributes significantly to glenohumeral stability.^9^^,^^11^ Notably, labral tears may lead to loss of the suction cup effect and labral resection has been shown to reduce the effectiveness of the concavity-compression mechanism by up to 20%.^9^^,^^14^ While it is plausible that the impact of a labral tear on anterior shoulder instability may depend on the underlying bony glenoid concavity, it remains unclear whether, in stable shoulders, the labrum compensates for reduced bony concavity. This approach is conceptually similar to observations in glenoid dysplasia, where posteroinferior bony deficiency is often associated with focal labral hyperplasia, suggesting a soft tissue adaptation to underlying bony insufficiency.^1^^,^^5^ Analogous to the BSSR which quantifies bony glenoid concavity, we introduce the labral shoulder stability ratio (LSSR) in this study as novel parameter, derived from labral and osteochondral measurements to provide a mathematical approximation of the chondrolabral contribution to glenoid concavity.

This study was designed to investigate whether a compensatory relationship exists between the labral morphology and the glenoid bone concavity. We hypothesized that patients with lower BSSR values (ie, flatter glenoids) would exhibit increased LSSR values, indicating compensatory deepening of the labrum to preserve stability.

## Methods

### Study design

We conducted a retrospective observational imaging study including 36 consecutive patients between January 2020 and December 2024, who underwent shoulder arthro–computed tomography scans for indications unrelated to instability. Inclusion criteria were as follows: (1) age between 18 and 60 years; (2) no history of ipsilateral shoulder dislocation or subluxation, confirmed by patient history and clinical examination; (3) no prior ipsilateral shoulder or glenoid fracture; (4) no prior ipsilateral shoulder surgery; and (5) no evidence of signs of instability on imaging such as labral tears, bony Bankart lesions or Hill-Sachs lesions. Ethical committee approval was obtained for this study (IRB-SOFCOT-Référence16-2025).

### CT-acquisition

After radiograph-guided intra-articular injection of radiographic contrast, high-resolution CT scans of the shoulder were acquired, using standardized imaging parameters: slice thickness <1.2 mm, number of slices >200, field of view covering the glenohumeral joint, X-Y resolution <0.5 mm, matrix size 512 × 512, 140 kV, and mA > 300. Scans were performed with the arm positioned in neutral rotation at the side. All images were anonymized and exported in DICOM (Digital Imaging and Communications in Medicine; DICOM, Danville, CA, USA) format for analysis using Horos medical image viewer (version 4.0.1; Horos Project, Geneva, Switzerland)

### BSSR and LSSR measurement

**Figure 1:**
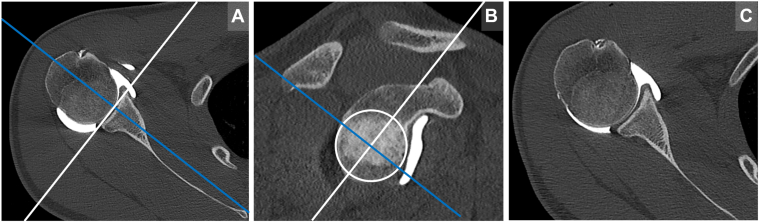
3D multiplanar reconstruction (MPR) of CT-imaging for standardized slice orientation and measurement reproducibility. (**A**) By adjusting the axial view axes perpendicular to the glenoid face and parallel to the scapular body, a strict sagittal plane aligned to the glenoid articular surface was selected. (**B**) In this sagittal plane, a best-fit circle was applied to the contour of the inferior glenoid. A line through the center of this circle and the long axis (*white line*) of the glenoid was determined. The plane perpendicular to this axis defined the standardized axial imaging plane (SAIP; *blue line*). (**C**) The axial CT slice in the SAIP used for subsequent measurements. *3D*, three-dimensional; *CT*, computed tomography; *SAIP*, standardized axial imaging plane.

**Figure 2:**
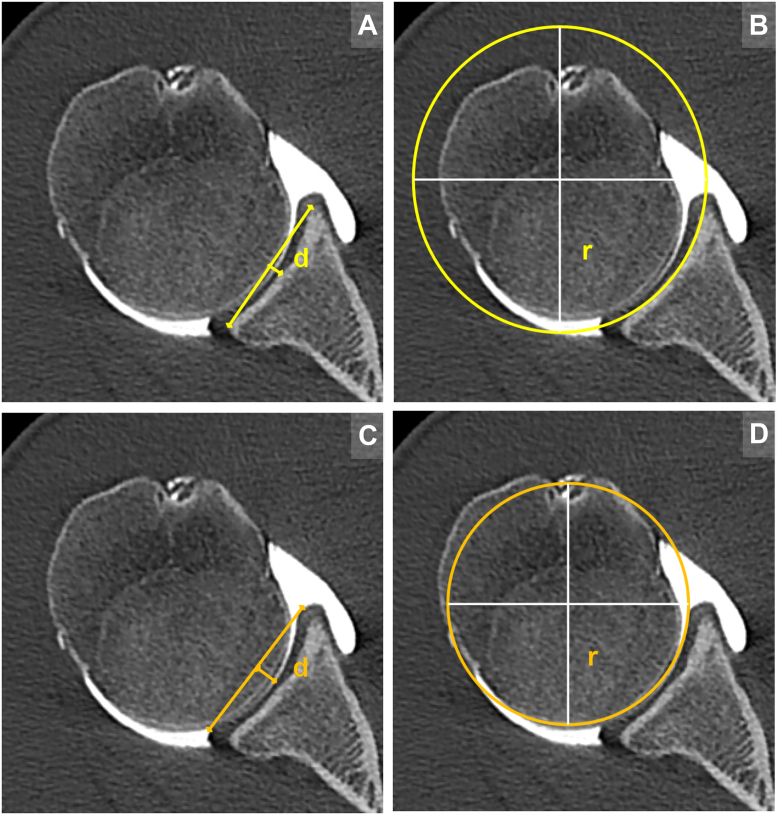
Measurement protocol performed in the standardized axial imaging plane (SAIP) to calculate the bony shoulder stability ratio (BSSR; **A** and **B**) and the labral shoulder stability ratio (LSSR; C-D). (**A**) Bony concavity depth (d): A straight line (*apical line*) was drawn between the anterior and posterior apices of the bony glenoid concavity. The depth was defined as the perpendicular distance from this apical line to the deepest point of the bony concavity. (**B**) Bony concavity radius (r): A best-fit circle was applied to the contour of the bony glenoid concavity. The radius of this circle represents the bony concavity radius. (**C** and **D**) Labral concavity depth and radius: Analogous to the bony concavity, the apical line was drawn across the labral contour. The labral depth was defined as the perpendicular distance from this line to the deepest point of the labrum and cartilage. A best-fit circle was also applied to this combined contour, and its radius represents the labral concavity radius (r).

All measurements were performed using the three-dimensional multiplanar reconstruction (MPR) functionality to ensure optimal slice orientation and reproducibility. First, a strict sagittal plane aligned to the glenoid face was selected by adjusting the axial view axes perpendicular to the glenoid face and parallel to the scapular body. Subsequently, the standardized axial imaging plane was determined, as described by Moroder et al. This plane was defined as perpendicular to the long axis of the glenoid, passing through the center of a best-fit circle (BFC) placed on the inferior portion of the glenoid in the sagittal view as depicted in Figure 1. In this plane, the concavity depth (d) and concavity radius (r) were measured as illustrated in Figure 2. Using these measurements, the BSSR was calculated with the following formula: BSSR = √{[1 - ((r - d)/r) ^2^]/[(r - d)/r]}.

Similarly, labral measurements were conducted to obtain the LSSR. The same methodology was applied, but measurements were based on the osteochondral surface, incorporating the labral morphology rather than the purely bony configuration as illustrated in Figure 2. The LSSR was calculated using the identical formula: LSSR = √{[1 - ((r - d)/r) ^2^]/[(r - d)/r]}

All measurements were independently performed by 2 experienced orthopedic shoulder surgeons, and the mean of their measurements was used for analysis to ensure reliability and minimize bias.

### Statistical analysis

All statistical analyses were performed using GraphPad Prism (version 10.3.1; GraphPad, San Diego, CA, USA). The inter-rater agreement between the 2 measurers for BSSR and LSSR was assessed using Bland-Altman analysis, with bias and limits of agreement reported. In addition, Pearson correlation coefficients (r) and corresponding *P* values were used to quantify the linear agreement between the 2 measurers. For each measured parameter, the mean of the 2 independent measurers' values was used in the analysis to ensure reliability. Descriptive statistics were computed for all continuous variables, including means, standard deviations, and ranges. To investigate the direct relationship between the parameters of interest, Pearson correlation analyses were performed as data was normally distributed as assessed by Shapiro-Wilk testing. Correlation results are presented as R^2^ values and Pearson r-values and 95% confidence intervals (CIs) are reported. For subgroup analysis, patients were stratified into low BSSR (≤25th percentile) and high BSSR (≥75th percentile) groups, and their respective LSSR values were compared. Independent sample t-tests were used to assess differences in mean LSSR between groups. A significance level of *P* < .05 was considered statistically significant.

## Results

### Inter-rater agreement for BSSR and LSSR

The Bland-Altman analysis revealed a mean bias of −0.014 for BSSR and −0.001 for LSSR, indicating no significant systematic difference. The limits of agreement ranged from −0.19 to 0.16 for BSSR and from −0.18 to 0.18 for LSSR, suggesting good overall agreement with minimal variability. Pearson correlation analysis confirmed the significant agreement between the 2 measurers for both BSSR (r = 0.72; *P* < .001) and LSSR (r = 0.75; *P* < .001).

### Concavity depth and best-fit-circle radius

A total of 36 patients were included in the analysis, comprising 20 right shoulders (55.6%) and 24 males (66.7%). The mean age was 26.7 ± 7.0 years (range: 19-45) and 24 (66.7%) were male. The mean bone concavity depth of the glenoid was 1.6 mm ± 0.8 mm (range: 0 mm-4.0 mm) and the labral concavity depth averaged 5.5 mm ± 1.0 mm (range 2.8 mm-7.3 mm). The mean radius of the BFC adhering to the glenoid bone concavity was 48.7 mm ± 16.2 mm (range: 24.2 mm-90.0 mm) and that of the labrum averaged 26.3 mm ± 3.6 mm (range: 19.4 mm-39.3 mm).

**Figure 3:**
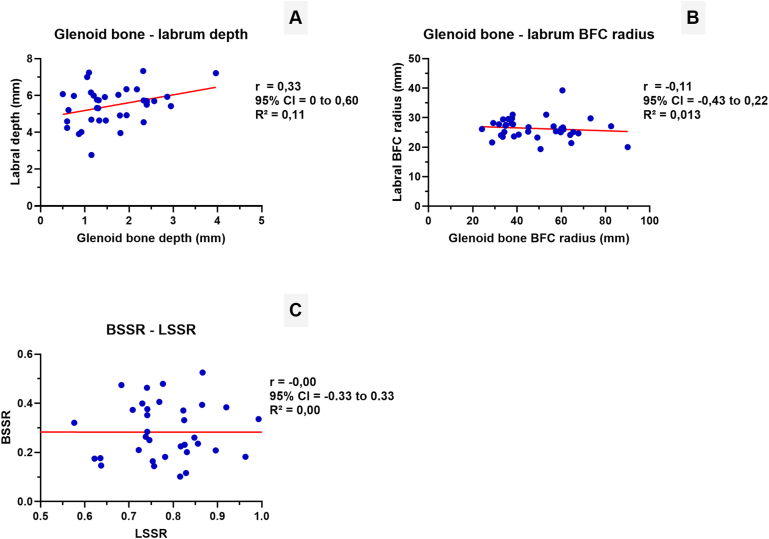
Scatterplots showing the relationship between the glenoid bone and labral depth (**A**), bone best-fit circle (BFC) radius and labral BFC radius (**B**) and between the bony shoulder stability ratio (BSSR) and labral shoulder stability ratio (LSSR). Each subplot displays a linear regression line with corresponding Pearson's correlation coefficient (r) and coefficient of determination (R^2^) values. There is a low positive correlation (r = 0.33; *P* = .049) between glenoid concavity depth and labral depth and no significant correlations between the bony or labral BFC radius nor between the BSSR and LSSR, indicating that labral morphology does not compensate for reduced bony glenoid concavity in stable shoulders.

There is a low positive correlation (r = 0.33; 95% CI 0-0.60; *P* = .049) between glenoid concavity depth and labral depth which contradicts that a flatter glenoid (lower bone depth) is compensated by deeper labral depth. There is no significant relationship between the radius of the glenoid bone and labrum (r = −0.11; 95% CI −0.43 to 0.22; *P* = .515) (Fig. 3).

### BSSR and LSSR

The BSSR had a mean value of 28.3% ± 11.1% (range: 10.2%-52.5%). The LSSR demonstrated a mean value of 77.9% ± 10.8% (range: 49.1%-100%). No significant correlation was observed between BSSR and LSSR (r = 0.01, 95% CI -0.33-0.33; *P* = 1.000), suggesting that glenoid flattening was not significantly associated with compensatory increases in labral concavity.

### Most flattened glenoids vs. most concave glenoids

To evaluate whether patients with a lower BSSR exhibit a compensatory increase in LSSR, we performed a subgroup analysis. Patients were divided into low BSSR (≤25th percentile) and high BSSR (≥75th percentile) groups, and their corresponding LSSR values were compared. The mean LSSR in the low BSSR group was 78.8%, while the mean LSSR in the high BSSR group was 75.9%. Statistical comparisons revealed no significant difference between the 2 groups (*P* = .554).

## Discussion

This study demonstrates that there is no compensatory relationship between the labral morphology and the glenoid bone concavity in patients without shoulder instability complaints, thus contradicting our hypothesis. We found that patients with lower BSSR (flatter glenoids) do not demonstrate a significant increase in chondrolabral concavity, suggesting that there is no systematic compensation for decreased bony glenoid concavity. Furthermore, there was a positive correlation between the bone concavity depth and labral concavity depth, further contradicting that a flatter glenoid is compensated by deeper labral depth.

The concept of BSSR, a quantifiable measure reflecting the relationship between glenoid concavity depth and the radius of a BFC approximating the humeral head, was introduced by Moroder et al^16^ in 2015. This ratio captures the stabilizing contribution of the glenoid's bony morphology and has since been validated as an important determinant of shoulder stability. In their study, patients with unilateral anterior instability demonstrated significantly lower BSSR values on their healthy shoulder (17.9% ± 8.5% in atraumatic and 23.9% ± 8.5% in traumatic instability groups) compared to a bilaterally healthy controls (31.1% ± 7.5%).^16^ These control values align with the mean BSSR observed in our study (28.3% ± 11.1%), confirming that the bony glenoid morphology in our cohort is representative of a stable shoulder population. In addition, Moroder et al^15^^,^^16^ reported a significantly lower glenoid bone depth in instability patients (1.1 mm ± 0.5 mm) compared to their control group (2.0 mm ± 0.5 mm), with the latter group aligning closely to our cohort's average bone depth (1.6 ± 0.8 mm). Their findings affirm that anterior shoulder instability is often associated with a reduction in glenoid depth which contributes to a lower BSSR.

Analogous to the findings of critical volumetric glenoid bone loss, Park et al^20^ demonstrated that glenoid concavity is strongly associated with surgical failure after arthroscopic stabilization for recurrent anterior shoulder instability. In their cohort of 120 patients, those who experienced recurrence had a significantly lower mean BSSR (18.6% ± 19.4%), and BSSR remained an independent predictor of failure in multivariate analysis (odds ratio 0.85; *P* = .02). Notably, they identified a critical BSSR threshold of 29.3%, below which patients exhibited a markedly increased risk of postoperative recurrence after soft tissue stabilization.^20^ The critical threshold closely approximates the mean BSSR values reported in both Moroder's healthy control group and our current cohort of stable shoulders.^16^ This may suggest that a greater proportion of instability patients may be at risk of recurrence after arthroscopic stabilization than would be expected based on volumetric bone loss alone. Accordingly, these findings highlight the need for further research relating to the incorporation of concavity analysis in addition to volumetric assessments for preoperative risk stratification when deciding on soft tissue stabilization or bony reconstruction.^2^^,^^3^^,^^18^^,^^24^ Conversely, recent work by Fury et al has demonstrated that bony surgical reconstruction techniques can restore or enhance glenoid concavity after glenoid bone loss. In a cadaveric model, they reported mean postoperative BSSR values of 45% for distal tibia allografts and 35% for Latarjet reconstructions, indicating a substantial improvement in glenoid concavity.^6^ Similarly, glenoid reconstruction with J-bone grafting typically overcorrects concavity loss, however at 1-year follow-up the glenoid morphology closely resembles the unaffected side due to remodeling.^17^

The glenoid concavity is not solely defined by its bony architecture as the glenoid labrum and cartilage cocontribute to and enhance the overall concavity of the glenoid fossa.^22^^,^^23^ In 2021, Wermers et al^23^ introduced the osteochondral shoulder stability ratio, which incorporates both bony and cartilaginous structures. Their directional analysis demonstrated that osteochondral shoulder stability ratio values were consistently higher than those of the BSSR, and that concavity varied depending on the axis of measurement. These findings support the notion that the chondral tissues meaningfully augment glenoid concavity. Furthermore, this provides a rationale for investigating whether the cartilage and to a further extent the labrum, quantified by the LSSR, might compensate for reduced bony concavity in stable shoulders.

An analogous concept has been described in the hip joint. In patients with acetabular dysplasia, Kraeutler et al^12^ reported a compensatory increase in labral size in response to deficient bony coverage. As a result, the total osseolabral coverage remained similar between dysplastic and anatomically normal hips, as measured by the center-edge angle. However, when applying the LSSR in our study, it is important to note that the mathematical relationship within the formula confirms that an increase in labral size or depth does not necessarily translate into a higher stability ratio, as the radius component acts as a counterbalancing factor. If labral compensation were occurring, we would expect a strong inverse relationship, namely, a larger glenoid radius (flatter bony concavity) being associated with a smaller labral radius (sharper labral curvature). This inverse relationship, however, was not observed in our data, suggesting that labral morphology does not adaptively compensate for reduced bony concavity in stable shoulders.

Moreover, while BSSR reflects the stability contribution of bone, which is rigid and maintains its geometry until structural failure, the LSSR represents the contribution of labral and cartilaginous tissue. These are more compliant and deformable under relatively low loading forces.^4^^,^^21^ As such, the actual mechanical stability provided may be substantially lower than the theoretical value suggested by the LSSR. This distinction is important as it highlights that even a structurally well-formed labrum may not effectively counteract humeral translation if its compressibility limits the capacity to resist shear forces. Further of note, the high variability in LSSR (range: 49.1%-100%) in our cohort, suggests that a similar labral tear may not implicate the same severity in terms of reccurence risk in individuals.

This study has several limitations. First, it is a retrospective imaging study with a relatively small sample size. However, the sample size was based on the feasibility and availability of eligible CT arthrograms during the study period. A post hoc power analysis confirmed that with 36 patients, the study was adequately powered (>75%) to detect a moderate correlation (r = 0.4) at a significance level of 0.05 between BSSR and LSSR. Second, while all included patients had no history of shoulder dislocation or subluxation, confirmed through patient history and clinical examination, they did undergo arthro-CT imaging for other clinical indications such as shoulder pain or scapulothoracic dyskinesia. As such, the cohort does not represent a completely asymptomatic or “healthy” population. Nevertheless, the measured glenoid bone depth and BSSR values closely matched those of control groups from prior studies, supporting the validity of the sample as a stable shoulder cohort. Third, recent work by Karpinski et al^10^ has shown that concavity-based measurements such as BSSR may benefit from automated measurement techniques, as manually performed assessments can exhibit only moderate reliability. Although our study used manual measurements, special attention was given to optimizing accuracy by performing all evaluations in the standardized axial imaging plane using multiplanar reconstructions as described. Furthermore, inter-rater agreement testing demonstrated good reliability with minimal systematic differences, supporting the robustness of the measurement protocol. Fourth, the LSSR is calculated from measurements of labral and cartilaginous structures obtained from CT arthrograms. These soft tissue structures may be subject to deformation during instability episodes, but in this study, they were assessed in their undeformed state. As such, dynamic changes were not captured in this present study. Moreover, our cohort consisted exclusively of clinically stable shoulders, which may have limited the variability in BSSR and LSSR values. As a result, potential compensatory relationships between these parameters in patients with anterior instability may not have been observable. It remains possible that in populations with greater or pathologic morphological variation, different patterns could emerge.

## Conclusion

This study demonstrates that labral morphology does not compensate for reduced bony glenoid concavity in clinically stable shoulders. Contrary to our hypothesis, lower BSSR was not associated with increased labral concavity, and no inverse relationship was observed between bone and labral curvature.

## Disclaimers

Funding: No funding was disclosed by the authors.

Conflicts of interest: Dr. Werthel serves as a consultant for Stryker. Dr. Moroder is consultant for Arthrex, Medacta and Alyve Medical. The other authors, their immediate families, and any research foundation with which they are affiliated have not received any financial payments or other benefits from any commercial entity related to the subject of this article.
